# The Establishment of a Novel Murine Model of Immune Thrombocytopenia in Pregnancy and the Impacts of Thrombopoietin Receptor Agonist on Platelet Production

**DOI:** 10.7759/cureus.71385

**Published:** 2024-10-13

**Authors:** Satoshi Shibata, Kohei Kitada, Kensaku Nakai, Ryo Uemura, Yasushi Kurihara, Mie Tahara, Akihiro Hamuro, Akemi Nakano, Takuya Misugi, Daisuke Tachibana

**Affiliations:** 1 Obstetrics and Gynecology, Osaka Metropolitan University Graduate School of Medicine, Osaka, JPN; 2 Obstetrics and Gynecology, Izumiotsu Municipal Hospital, Izumiotsu, JPN; 3 Obstetrics and Gynecology, Osaka City General Hospital, Osaka, JPN

**Keywords:** immune thrombocytopenia, itp murine model, pregnancy, romiplostim, thrombopoietin receptor agonist (tpo-ra)

## Abstract

Objective

Immune thrombocytopenia (ITP) is frequently associated with pregnancy. However, treatment options for ITP in pregnancy are limited, and there are few animal models available for the establishment of treatments. Here, we aimed to establish a novel murine pregnant model of ITP and to investigate the impacts of thrombopoietin receptor agonist (TPO-RA) on platelet production and reproductive outcomes.

Methods

Anti-glycoprotein Ib-alpha (GPIbα) antibody, which binds to megakaryocytes and platelets, was subcutaneously administered to pregnant mice in order to develop an ITP model (ITP group). TPO-RA was given in doses of 1 µg/kg, 10 µg/kg, and 100 µg/kg (low-dose group, mid-dose group, and high-dose group, respectively) for the treatment of ITP in pregnancy.

Results

The ITP group showed a significant reduction of platelet counts of less than 15% of healthy pregnant mice (control group) and also showed a significant increase in miscarriage rate (control group, 3.8%; ITP group, 44.4%; p < 0.05). Striking increases in platelet counts were observed in every TPO-RA group without any negative effects on fetal growth and placental pathology. No abnormality was noted in the external examination of fetal mice. Interestingly, a significant recovery of miscarriage rate was observed in the mid-dose group (23.5%) compared with the ITP group (p < 0.05).

Conclusion

A novel ITP model in pregnant mice was induced by injection of an anti-GPIbα antibody, and sufficient effects of TPO-RA on platelet production were observed in the present study. Furthermore, the positive impacts of TPO-RA on reproductive outcomes were revealed in the ITP model.

## Introduction

Immune thrombocytopenia (ITP) is an autoimmune disease characterized by low platelet counts and mucosal bleeding. The estimated incidence rate of ITP in adults is 5-10/100,000, and the pathophysiological mechanism is thought that antiplatelet antibodies, such as glycoprotein Ib-alpha (GPIbα) and glycoprotein IIb/IIIa (GPIIb/IIIa), bind directly to platelets, leading to the hyperdestruction of platelets in the spleen and the liver and these antibodies also bind to megakaryocyte, resulting in impaired platelet production [1]. To date, the recommended first-line therapy for adult ITP patients is corticosteroids or intravenous immunoglobulin (IVIG), while monoclonal antibody treatment or thrombopoietin (TPO) receptor agonist (TPO-RA) has been used as a second-line therapy [2].

The incidence rate of ITP in pregnant women is 10-100/100,000, which is relatively higher compared to non-pregnant women. Approximately one-third of pregnant women with ITP need treatments for low platelet counts [3]. The treatment for pregnant ITP patients is considered when the platelet counts drop below 20-30 × 10^9^ /L, and prompt treatments are required at the timing of bleeding, surgical intervention, and delivery [4]. The same first-line therapy used for adults with ITP is also applied to pregnant women with ITP, although it might be less effective during pregnancy compared with non-pregnant adults [5]. TPO-RA has been established as one of the second-line therapy for adults with ITP, although its clinical use for pregnant women is limited [6]. Because of those reasons, management of ITP patients during pregnancy remains to be still challenging.

Due to the difficulties in treating ITP in pregnancy, it is crucial to develop an appropriate animal model to explore potential treatment. Liu et al. previously established a murine model of ITP in pregnancy by administering anti-GPIIb/IIIa antibody and showed efficacy and safety of recombinant human TPO (rhTPO) [7]. However, induction of ITP in pregnant mice with anti-GPIIb/IIIa antibody is relatively complicated since frequent injection in different doses is necessary, and the reproductive outcome was not elucidated. Furthermore, rhTPO is now rarely used due to the side effect of the production of neutralizing antibodies against TPO, leading to more severe thrombocytopenia [8]. Anti-GPIbα antibody is also used to induce ITP in mice with fewer injections compared to anti-GPIIb/IIIa antibody; however, there are no reports of its administration to pregnant mice [9].

Recently, we have reported that TPO-RA has the striking effect of increasing platelet production without any negative impacts on reproductive outcomes in normal pregnant mice [10]. However, in our previous study, TPO-RA was administrated to only normal pregnant mice, and the effects of TPO-RA on pregnant mice with low platelet counts are still unclear. In this study, we aimed to establish a novel murine model of ITP in pregnancy with an anti-GPIbα antibody. We also intend to elucidate the effects of TPO-RA on this animal model, including reproductive outcomes.

## Materials and methods

Animal model

This study was conducted from December 5, 2022, to March 31, 2024. All research protocols and experimental methods were aligned with the standards of the Osaka Metropolitan University Animal Experiment Committee and received ethical approval on December 5, 2022 (approval number: 22075). ICR mice aged eight to 12 weeks on the first day of pregnancy were obtained from SLC (Hamamatsu, Japan). The identification of a copulatory plug was used as confirmation of successful mating, marking that day as the first day of pregnancy. The welfare of all mice was prioritized, following the NIH’s Guide for the Care and Use of Laboratory Animals. Mice were maintained in a controlled environment with temperatures at 24 ± 1°C, humidity at 55 ± 5%, and alternating 12-hour light/dark cycles. They had constant access to water and standard rodent feed.

Induction of ITP in pregnant mice and treatment with TPO-RA

We developed a model of ITP in pregnant mice using a specific dosage of rat anti-GPIbα antibody (R300, Emfret Analytics GmbH & Co. KG, Wurzburg, Germany) adjusted according to the mouse's weight to cultivate a consistent model of platelet destruction. Specifically, pregnant ICR mice were administered subcutaneous injections of R300 at 4 µg/g every four days [9]. Romiplostim (Nplate®, Amgen; dosages of 1, 10, and 100 µg/kg), which is known as TPO-RA, was administered subcutaneously on gestational days (GDs) 1, 8, and 15 for the purpose of increasing platelets [2]. The healthy pregnant mice were classified as control group (n = 4), the mice that received only R300 were categorized as ITP model group (n = 5), and those treated with R300 were divided into three groups according to the dosage of romiplostim (1 µg/kg, 10 µg/kg, and 100 µg/kg as low-dose (n = 3), mid-dose (n = 3), and high-dose (n = 3) groups, respectively).

Sample and tissue preparation

A blood sample of 6 µL was collected from the lateral tail vein of pregnant mice using a 25-gauge needle and micropipette coated with ethylenediaminetetraacetic acid (EDTA). Those blood samples were stored in microtubes mixed with EDTA and 30 µL of phosphate-buffered saline (PBS). Blood cell counts were conducted within an hour using an automated animal hematology analyzer, MEK-6450 (Nihon Kohden Corporation, Tokyo, Japan). Mice were euthanized under isoflurane anesthesia (1.5-3% inhalation) through cervical dislocation in order to collect femoral bones, fetal blood, fetal liver, and placentas. Measurements, such as miscarriage rate and fetal and placental weights at birth, were also documented. Fetal blood was collected using a micropipette after puncturing the anterior facial vein with a 25-gauge needle, gathering 10 µL into microtubes filled with EDTA in PBS. Blood cell counts were then assessed [10].

Histology

Placenta and fetal liver tissues were fixed in formalin, embedded in paraffin, and sectioned into 4 µm slices. For morphological analysis, the sections were stained with hematoxylin and eosin (H&E). The femoral bone of pregnant mice was decalcified with Decalcifying Solution A (Fujifilm, Osaka, Japan) for 12 hours, then embedded in paraffin and stained with H&E [10].

Counting of megakaryocytes 

Megakaryocytes are significantly larger than red blood cells at 10 to 15 times their size, with diameters of 50 to 100 µm and a conspicuous, migrating nucleus, allowing for easy visual inspection and counting under a microscope. The frequency of megakaryocytes on each slide was determined by averaging the number of megakaryocytes per field (the total number of megakaryocytes divided by the total number of fields inspected) [10].

Statistical analysis

Data were presented as medians and ranges. Comparisons between groups were made using the Mann-Whitney U test or the Student’s t-test. A p-value of less than 0.05 was considered statistically significant, and analyses were performed using SPSS version 21 software (IBM SPSS Statistics for Windows, IBM Corp., Armonk, NY).

## Results

Effects of anti-GPIbα antibody (R300) on platelet counts in pregnant mice

**Figure 1:**
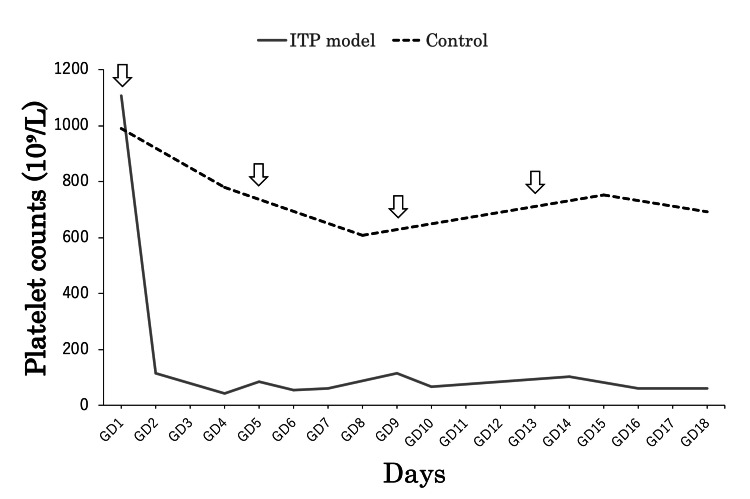
Effect of TPO-RA on platelet counts in pregnant ITP model mice and its fetuses Changes of platelet counts in pregnant mice during antibody administration. The data are expressed as platelet counts (×10^9^ /L; mean). We injected R300 to establish the ITP model and injected the same volume of PBS as a control on GDs 1, 5, 9, and 13. Arrows show the days when we injected R300 or PBS. As shown, the platelet counts of the ITP model group decreased to an extremely low level on GD 2 and were kept at a significantly lower level during pregnancy (p < 0.05). GD, gestational day; ITP, immune thrombocytopenia; PBS, phosphate-buffered saline; TPO-RA, thrombopoietin receptor agonist

The changes in platelet counts in healthy pregnant mice and pregnant mice with R300 injection are shown in Figure 1. In pregnant mice treated with R300 on GD 1, the platelet counts decreased sharply and significantly to the lowest levels on GD 2 and remained low until GD 18 with every-four-days administration, when compared with the control group (p < 0.05), indicating the successful establishment of an ITP pregnant murine model. Petechiae, swelling, or persistent bleeding were not observed at the injection sites of PBS or R300 in any mice.

Effects of TPO-RA (romiplostim) on platelet counts in pregnant ITP model

**Figure 2:**
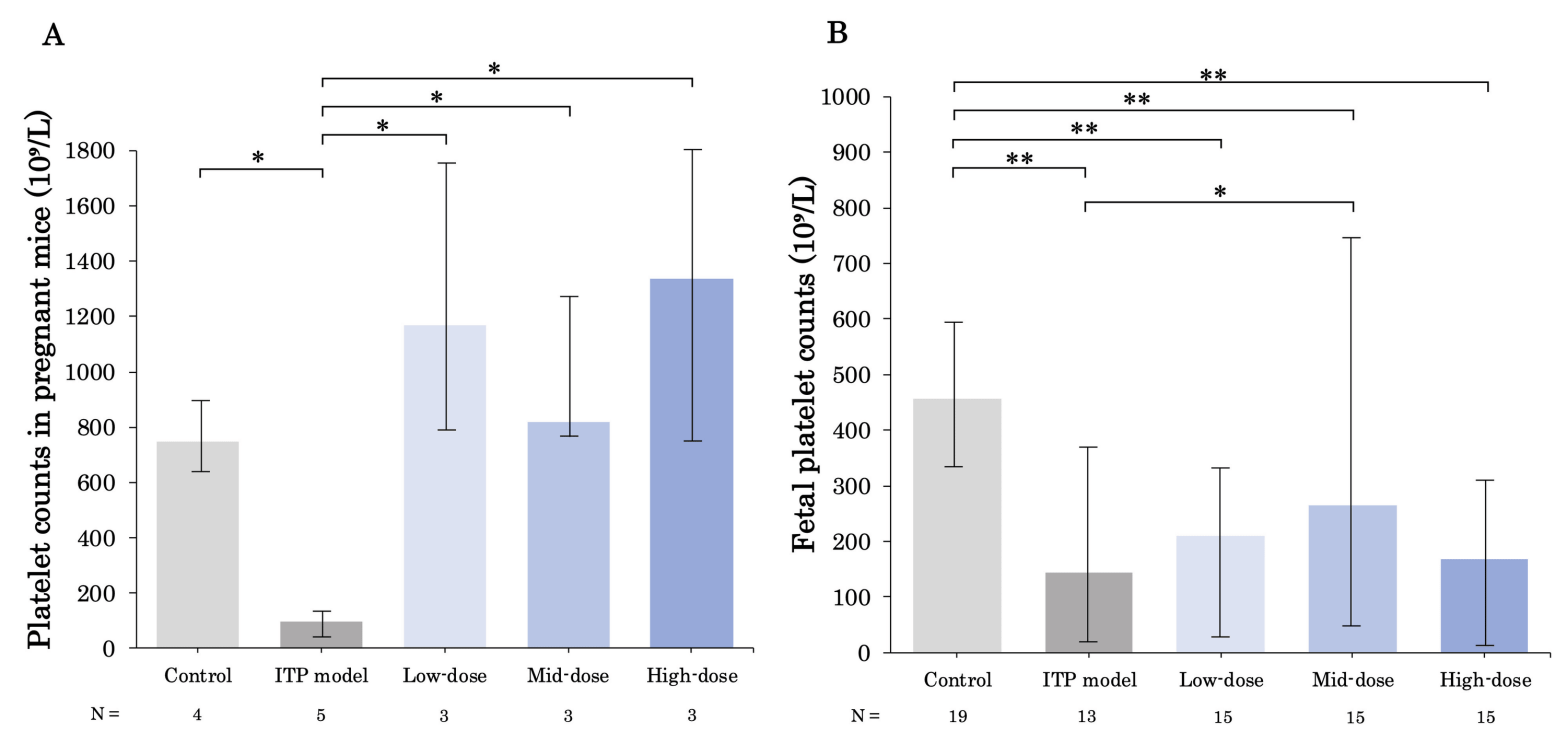
Platelet counts in pregnant mice during anti-GPIbα antibody (R300) administration Platelet counts in each pregnant group on GD 14 (A) and in each fetus at birth (B). R300 significantly declined both maternal and fetal platelet counts, and romiplostim significantly increased maternal platelet counts of ITP model pregnant mice. In addition, Figure 2B shows a significant increase in fetal platelet counts in the mid-dose group compared to that of the ITP model group. Compared to the control group, fetal platelet counts significantly declined in R300 groups. Bars represent the median. Error bars represent the range. * p < 0.05, ** p < 0.01. GD, gestational day; GPIbα, glycoprotein Ib-alpha; ITP, immune thrombocytopenia

Figure 2A shows a comparison of maternal platelet counts at GD 14 among all groups. The median platelet counts (×10^9 ^/L) at GD 14 for each group were 748, 96, 1,170, 818, and 1,338 (control group, ITP model group, low-dose, mid-dose, and high-dose group, respectively). The three groups that received romiplostim had significant increases in platelet counts compared to the ITP model group (p < 0.05 in each comparison). Figure 2B shows the platelet counts of the fetuses. Compared to the control group, the four groups treated with R300 had significantly reduced fetal platelet counts (×10^9^ /L): 456 (control group), 144 (ITP model group), 210 (low-dose group), 264 (mid-dose group), and 168 (high-dose group). p < 0.01 was observed in R300 received groups compared to the control group. Fetuses in the mid-dose group showed a significant increase in platelet counts compared to those in the ITP model group (144 in the ITP model group and 264 in the mid-dose group; p < 0.05), while the low-dose and high-dose groups did not show a significant increase.

Megakaryocyte counts in maternal and fetal tissues

**Figure 3:**
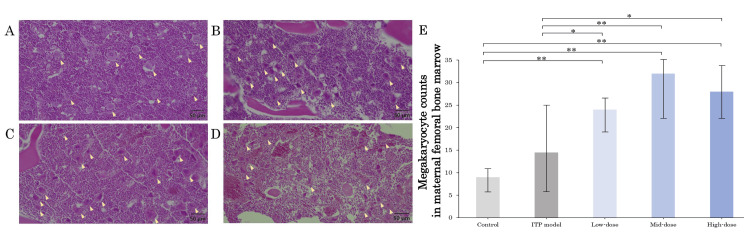
Histology of megakaryocytes of femoral bone marrow in each group Femoral bone marrow histology (H&E) on GD 18 in ITP model group (A), low-dose group (B), mid-dose group (C), and high-dose group (D). Megakaryocyte frequency (the total number of megakaryocytes divided by the total number of scorable fields) under 20 magnification is shown for all groups (E). R300 significantly increased megakaryocytes compared to the control group, and additionally, romiplostim significantly increased megakaryocytes compared to the ITP model group. The yellow arrowheads mark megakaryocytes. Scale bars: 50 µm, at 20× magnification. * p < 0.05, ** p < 0.01. GD, gestational day; ITP, immune thrombocytopenia

**Figure 4:**
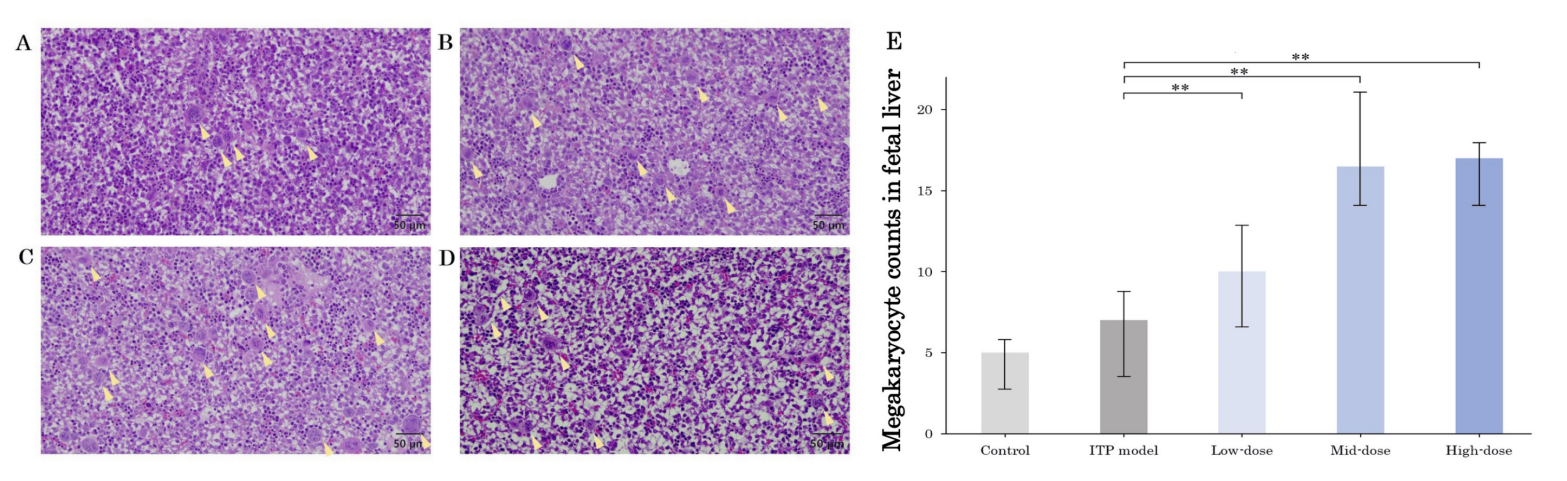
Histology of megakaryocytes of fetal liver in each group Histology (H&E) of megakaryocytes of the fetal liver in the ITP model group (A), low-dose group (B), mid-dose group (C), and high-dose group (D). The yellow arrowheads mark megakaryocytes. Figure 4E shows megakaryocyte frequency under 20 magnification is shown for all groups. The ITP model group showed no significant difference in megakaryocyte counts compared to the control group. On the other hand, romiplostim showed a significant increase in megakaryocytes compared to the ITP model group. Scale bars: 50 µm, at 20× magnification. Bars represent the median. Error bars represent the range. ** p < 0.01. ITP, immune thrombocytopenia

Figures 3A-3D demonstrate the H&E staining of the megakaryocytes in the maternal femoral bone marrow of the ITP model group and romiplostim-administered groups. There were significant increases in megakaryocytes in the ITP model, low-dose, mid-dose, and high-dose groups compared to the control group (Figure 3E). Figures 4A-4D show the HE staining of megakaryocytes in the fetal liver of the ITP model group and romiplostim-administered groups. There was no significant difference in megakaryocytes in the fetal liver in the ITP model group compared to the control group (Figure 4E). Three groups treated with romiplostim showed significant increases in megakaryocytes compared to that of the ITP model group (p < 0.01 was observed in three groups). No significant difference in megakaryocytes was observed among romiplostim-treated groups.

Effects of anti-GPIbα antibody (R300) and TPO-RA (romiplostim) on miscarriage rates 

**Figure 5:**
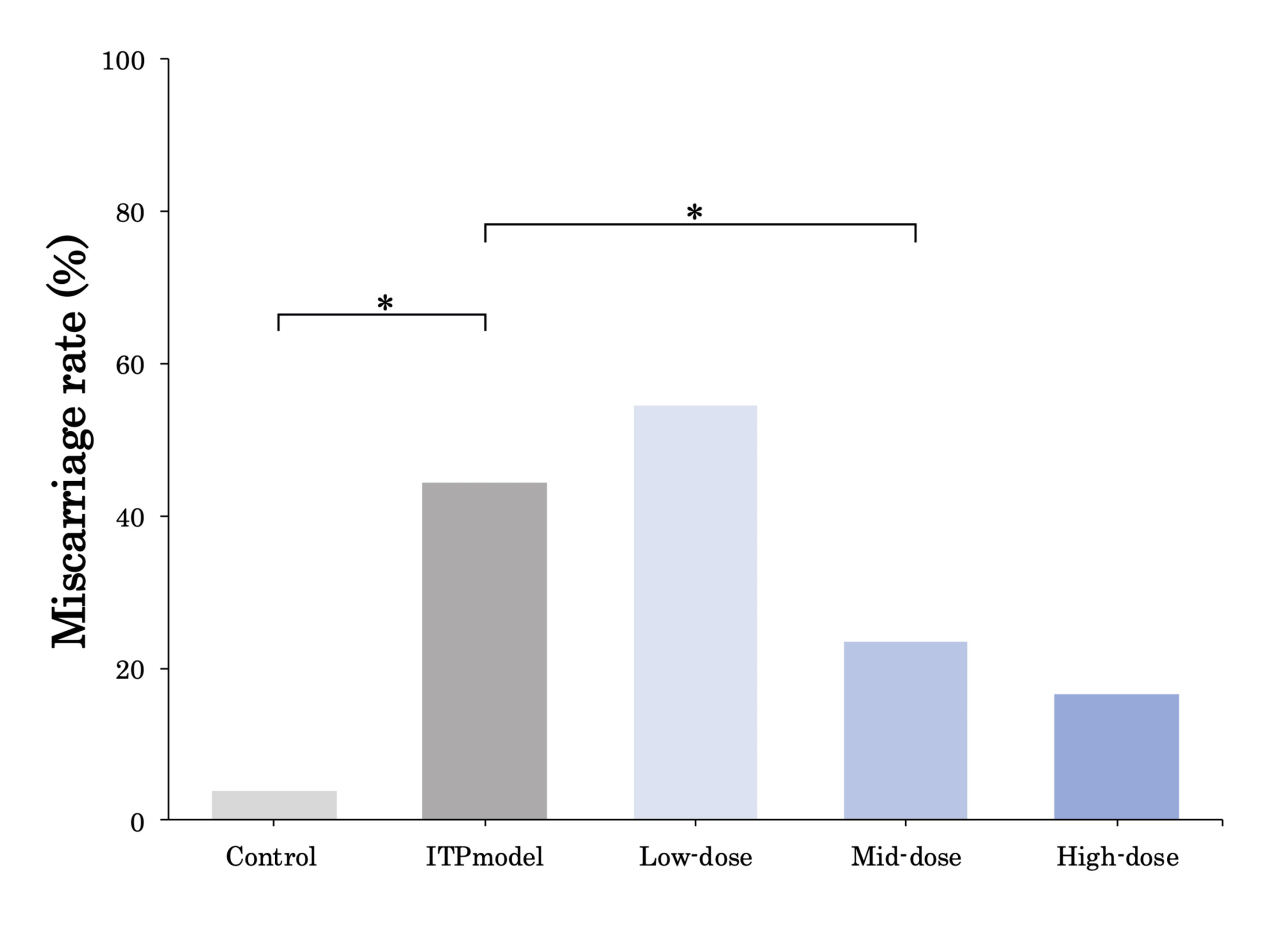
Effects of R300 and romiplostim on miscarriage rate Miscarriage rates in each group are shown. R300 significantly increased the miscarriage rate compared to the control group. Romiplostim showed no significant difference compared to the control group, but the miscarriage rate of the mid-dose group significantly reduced compared to that of the ITP model group. Bars represent the median. * p < 0.05. ITP, immune thrombocytopenia

Unexpectedly, as shown in Figure 5, a significant increase in miscarriage rates was observed in the ITP model group (44%) compared to the control group (3.8%). Even more surprisingly, a striking reduction in miscarriage rate was noted in the mid-dose group (23.5%) when compared to the ITP model group (p < 0.05).

Effects of anti-GPIbα antibody (R300) and TPO-RA (romiplostim) on fetal and placental growth

**Figure 6:**
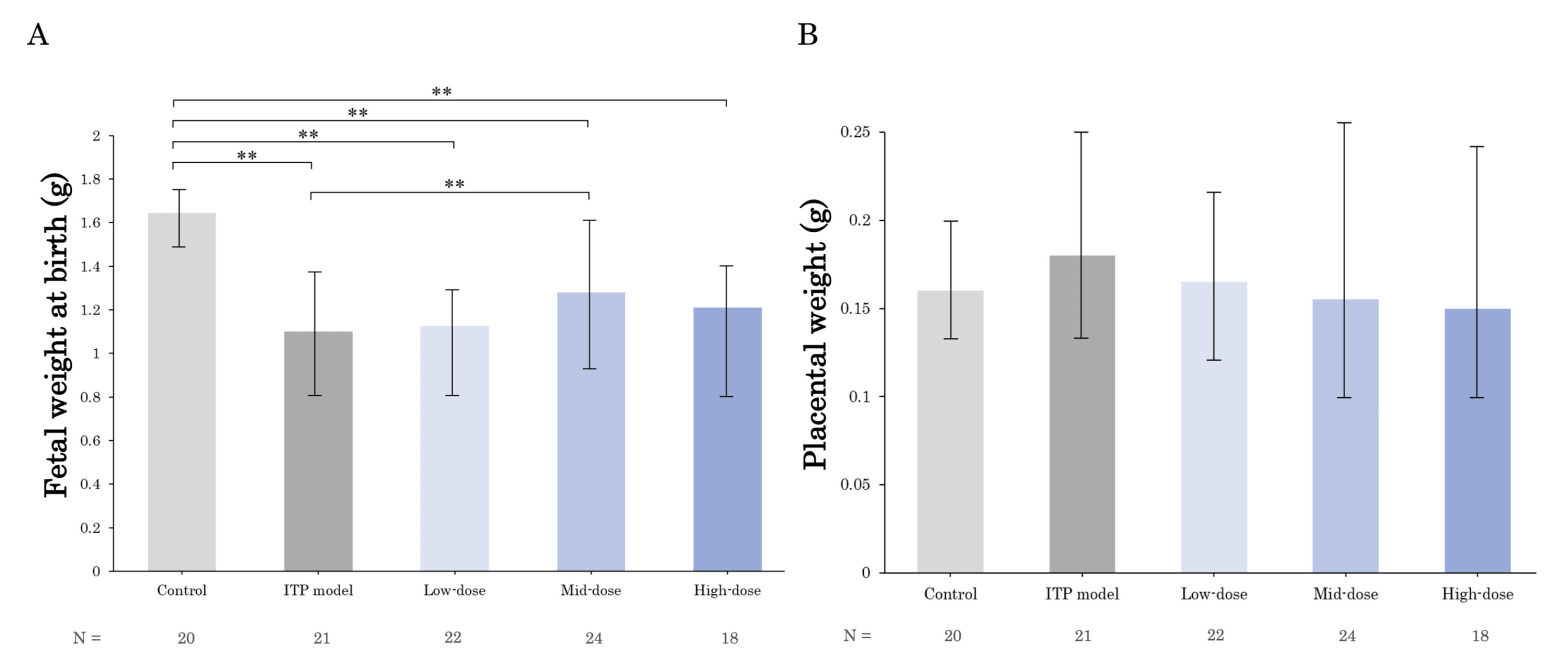
Effects of R300 and romiplostim on fetal weights and placental weights in each group Fetal weights (A) and placental weights (B) of each group are shown. Administration of R300 significantly reduced their weight compared to the control group. In the mid-dose group, romiplostim significantly improved fetal weight compared to the ITP model group. There was no significant difference in placental weight between each group. Bars represent the median. Error bars represent the range. ** p < 0.01. ITP, immune thrombocytopenia

Figure 6A shows the fetal weights of the groups. Compared to the control group, the ITP model, low-dose, mid-dose, and high-dose groups showed significant decreases in their fetal weight (p < 0.01). In comparison to the ITP model group, the mid-dose group showed a significant increase in fetal body weight (p < 0.01). The placental weights of each group are shown in Figure 6B. No significant change in placental weight was observed in each group.

## Discussion

In this study, we first established the ITP murine model during pregnancy using anti-GPIbα antibody, and we also demonstrated the efficacy and safety of TPO-RA during pregnancy. The phenomena of decreased platelets and increased megakaryocytes in the fetuses provide indirect evidence that anti-GPIbα antibodies and romiplostim cross the placenta. Furthermore, positive effects of TPO-RA on reproductive outcomes were suggested by the improvement of fetal growth and miscarriage rate.

TPO-RA binds to the TPO receptor and makes megakaryocytes produce platelets that are resistant to clearance, resulting in the prolongation of their circulation and an increase in their counts [11]. TPO-RA was approved for clinical use in 2008 and has been used as a second-line treatment for adult ITP. The US Food and Drug Administration (FDA) categorizes TPO-RA use during pregnancy as category C, and no clinical trials on pregnant humans have been conducted so far, although some authors have reported the efficacy of TPO-RA for pregnant women [12].

Methods to establish the ITP murine model are mostly classified into three types: induced ITP, secondary ITP, and passive ITP [13]. Induced ITP models are introduced by transfusion of heterologous platelets or transplanting splenic cells from platelet antigen-deficient mice, and then immune responses, where platelet antigens are recognized as foreign, subsequently develop [14,15]. Secondary ITP models are caused by the application of pathological mechanisms, such as lupus nephritis, infections, and damage caused by drugs. However, secondary ITP models develop not only to thrombocytopenia but also to severe microvascular injuries and cardiovascular events [16]. Passive ITP models seem mostly to mimic the pathophysiological mechanism of ITP by the administration of IgG anti-platelet antibody [17]. The prominent platelet decrease is achieved within 24 hours of the first IgG injection, and the low platelet counts can be maintained by repeated anti-platelet antibody administration. This passive model is thought to be an ideal model and is widely used in the research fields [18,19].

To our knowledge, there is one report as to the effects of rhTPO on a pregnancy ITP model, which was established by administering anti-GPIIb/IIIa antibody. The authors showed an increase in platelet counts and reported that there was no negative effect on fetal growth after birth [7]. Although they did not observe the rate of miscarriage or the development of the fetus and placenta, the theoretical miscarriage rate might have been increased due to placental malperfusion caused by antibody-induced activation and aggregation of platelets [20]. In fact, pregnant women with GPIb-IX deficiency and anti-GPIbα antibodies have been reported to be at high risk of miscarriage [21,22], and our ITP model really demonstrated that the rate of miscarriage significantly increased from 3.8% in the control group to 44% in the ITP group. The mechanism of impaired fetal growth and increased miscarriage rate by anti-platelet antibody should be further discussed, as well as the improvement mechanism of the phenomena by TPO-RA. 

In hematopoietic stem cells, such as megakaryocytes, the Janus kinase/signal transducer and activator of transcription (JAK-STAT) pathway is activated by TPO through the TPO receptor and then promotes differentiation into megakaryocytes, leading to an increase of platelet production [23]. Recent reports suggest the involvement of the JAK-STAT pathway in reproductive processes and outcomes. Recently, Segerer et al. found TPO receptors in the decidua of early pregnancy, and they demonstrated that the JAK-STAT activation through the TPO receptor contributes to setting up an optimal microenvironment for maternal-fetal immune tolerance [24]. Furthermore, Ding et al. have shown that mesenchymal stem cells, which are derived from human umbilical cords, can reduce the rate of miscarriage by regulating immune responses through Th1 and Th2 cell balance via the JAK-STAT pathway in a miscarriage animal model [25]. Taking these reports into consideration, the underlying mechanism of our observation that the administration of TPO-RA to ITP model mice improved the miscarriage rates might be possibly explained by the activation of the JAK-STAT pathway.

It is estimated that approximately 70-80% of adult ITP patients have anti-GPIIb/IIIa antibodies, and the other 20-40% of patients have anti-GPIbα antibodies. In addition, some patients may also have both antibodies or antibodies against other platelet surface glycoproteins [26]. Splenic macrophage is generally considered to destruct the platelets opsonized by anti-GPIIb/IIIa antibody through the Fc receptor [9]. On the other hand, the Ashwell-Morell receptor expressed on hepatocytes is thought to play substantial roles in the clearance of anti-GPIbα opsonized platelets in the liver [27]. These differences in platelet destruction manners depending on the types of anti-platelet antibodies might explain the reasons why IVIG therapy and/or splenectomy do not exhibit sufficient effect in the management of ITP. In fact, these clinical findings may be supported by the report from Mahevas et al. that combination therapy with TPO-RA increases the efficacy [28]. Furthermore, the physiological changes of normal pregnancy, such as the increased systemic circulation volume and the high perfusion of each organ, may attenuate the effectiveness of conventional treatments [29]. Considering these factors, the advent of TPO-RA is expected to be an evangel for pregnant women complicated with refractory ITP.

This study includes some limitations that have yet to be investigated. Firstly, we did not examine the precise mechanisms related to the increased miscarriage rate caused by the administration of anti-GPIbα antibodies. Secondly, we did not follow the long-term neonatal outcomes, including neurological development. Lastly, since this study is based on animal experiments, further research is needed before applying it to humans.

## Conclusions

In this study, we established a novel pregnant ITP murine model using anti-GPIbα antibody. Compared to another murine model of ITP in pregnancy, this model was able to be generated more simply and with fewer injections. We also demonstrated that TPO-RA treatment against ITP during pregnancy increased platelet counts and did not inhibit the development of the fetus and placenta in the murine model. Since treatments for ITP in pregnancy are limited, our research is expected to contribute to the establishment of new treatment options for ITP in pregnancy. Furthermore, TPO-RA recovered the miscarriage rate, which was shown in our pregnant ITP mice model. TPO-RA could have positive effects on not only low platelet counts but also obstetrical outcomes in pregnant women complicated with ITP.
